# Acral Melanoma: A Review of Its Pathogenesis, Progression, and Management

**DOI:** 10.3390/biom15010120

**Published:** 2025-01-14

**Authors:** Soo Hyun Kim, Hensin Tsao

**Affiliations:** 1 Harvard Medical School, Boston, MA 02115, USA; 2 Department of Dermatology, Wellman Center for Photomedicine, Massachusetts General Hospital, Harvard Medical School, Boston, MA 02114, USA

**Keywords:** acral melanoma, genetics, genomics, therapy

## Abstract

Acral melanoma is a distinct subtype of cutaneous malignant melanoma that uniquely occurs on ultraviolet (UV)-shielded, glabrous skin of the palms, soles, and nail beds. While acral melanoma only accounts for 2–3% of all melanomas, it represents the most common subtype among darker-skinned, non-Caucasian individuals. Unlike other cutaneous melanomas, acral melanoma does not arise from UV radiation exposure and is accordingly associated with a relatively low tumor mutational burden. Recent advances in genomic, transcriptomic, and epigenomic sequencing have revealed genetic alterations unique to acral melanoma, including novel driver genes, high copy number variations, and complex chromosomal rearrangements. This review synthesizes the current knowledge on the clinical features, epidemiology, and treatment approaches for acral melanoma, with a focus on the genetic pathogenesis that gives rise to its unique tumor landscape. These findings highlight a need to deepen our genetic and molecular understanding to better target this challenging subtype of melanoma.

## 1. Introduction

**Figure 1:**
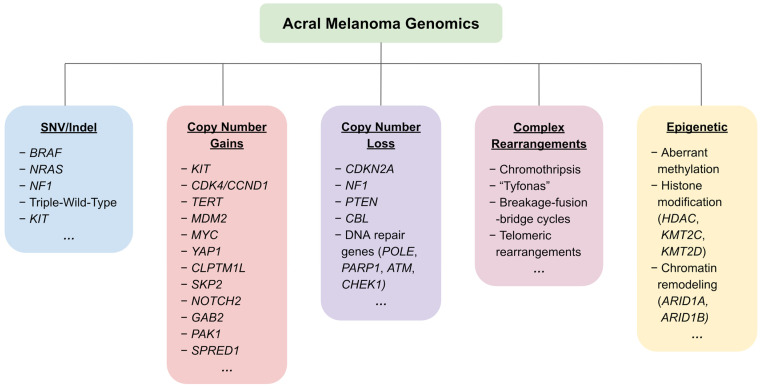
An overview of the most frequently reported genetic and epigenetic alterations in acral melanoma.

The incidence of cutaneous melanoma, the most fatal form of skin cancer, has been steadily rising globally. Despite recent advances such as targeted therapies for cutaneous melanomas, the genetic and molecular pathogenesis of acral melanoma (AM) is much less understood. Unlike other cutaneous melanomas, AM does not arise from UV radiation exposure, and its genetic and molecular landscape varies significantly from other melanoma subtypes. Recent advances in genomic, transcriptomic, and epigenomic sequencing have revealed genetic alterations unique to AM, including novel driver genes, high copy number variations, and complex chromosomal rearrangements (Figure 1). This review synthesizes the current knowledge on the clinical features, epidemiology, and treatment approaches for AM, focusing on the genetic pathogenesis that gives rise to its unique tumor landscape.

## 2. Clinical Features and Epidemiology

**Figure 2:**
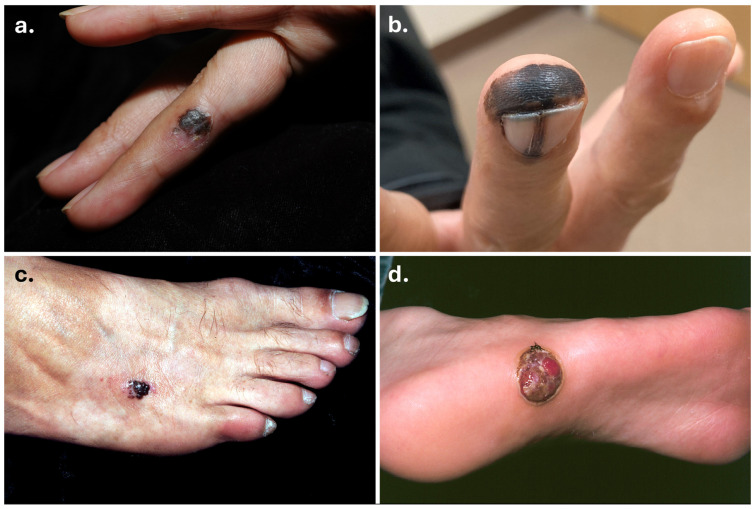
Clinical images of acral melanomas. Acral melanomas on the finger (**a**), fingertip and nail matrix (**b**) (courtesy of Dr. Arthur Tong), dorsum of foot (**c**), and plantar surface (**d**).

Acral melanoma (AM; Figure 2) is a distinct subtype of cutaneous melanoma (CM) that uniquely occurs on glabrous (non-hair-bearing) skin of the palms, soles, and nail beds that are UV-shielded [1]. AM clinically presents as a pigmented plaque or macule, sometimes nodular, with or without ulceration [1]. Acral lentiginous melanoma is the most common histopathological subtype that arises on acral sites, with “lentiginous” referring to the lateral expansion of atypical melanocytes at the dermoepidermal junction basale (radial growth phase) before invading the dermis [2,3]. AM is often characterized by a long radial growth phase that can take months to years before evolving into an invasive stage indicated by a nodular component [1,4]. Distinct genetic patterns have characterized melanomas arising on dorsal (included as AM by Haugh et al.), volar, and subungual/interdigital sites of acral skin [5], with subungual/interdigital AMs demonstrating higher rates of oncogenic mutations and copy number aberrations than dorsal or volar sites [5]. Subungual AM most commonly affects the great toe or the thumb, presenting as longitudinal melanonychia with multiple pigmented lines of varying thickness [1,6]. However, subungual AM can also present with diffuse pigmentation or ulceration without pigmentation [1,7].

While AM only accounts for 2–3% of all melanomas in Western populations, it represents the most common subtype among non-Caucasian individuals, namely those of African, Asian, and Hispanic descent [1]. The incidence of AM in the United States is 2.0 per million person-years [8]. The proportion of AM among all melanoma subtypes is greatest in Black Americans (33.6%), followed by Asian/Pacific Islanders (23.1%) and Hispanic Whites (9.3%), and is lowest in non-Hispanic Whites (1%) [8]. In Asian countries such as Taiwan, China, Japan, Korea, Hong Kong, and Singapore, AM has been reported to account for up to 58% of all melanomas [9].

AM is often diagnosed at later stages and tends to be associated with poor clinical courses, with 62% of AMs being diagnosed at stage II or above compared with 32% of CMs [1]. The 5-year melanoma-specific survival (MSS) rate of AM was significantly lower than that of overall CM (80.6 vs. 93.0%) [8]. The comparatively poorer prognosis may be due to AM being associated with an increased Breslow thickness due to delayed diagnosis, ulceration, and lymphovascular invasion at presentation [10,11]. Notably, Black Americans had the worst 5-year MSS rates (66.9%) amongst all racial and ethnic groups and tended to be diagnosed at more advanced stages [8,12].

The anatomical location of AM, which can involve subungual or digital regions, often makes achieving the recommended surgical excision margins challenging and can compromise function in the process [13]. Moreover, the unique genetic and immune landscape of AM has resulted in lower efficacies for emerging systemic therapies, such as BRAF/MEK inhibitors and immune checkpoint inhibitors (ICI).

## 3. Genetic and Genomic Aberrations in Acral Melanoma

**Figure 3:**
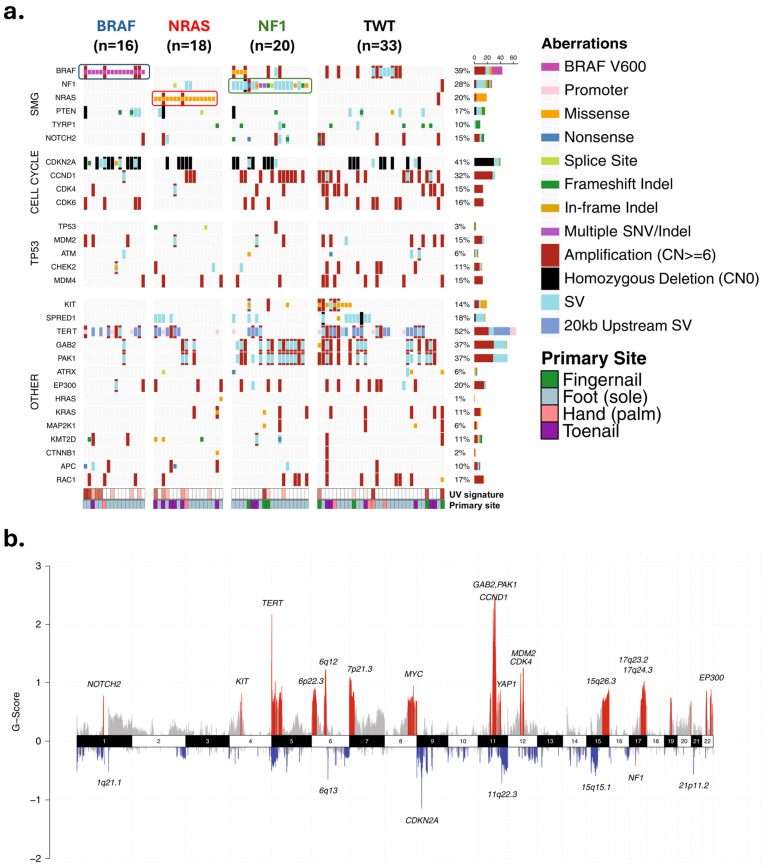
Landscape of genetic and genomic abnormalities in acral melanomas. (**a**) Mutations in different loci identified through whole genome sequencing of 87 acral melanomas. Tumors were from the soles (n = 59), palms (n = 6), and subungual regions (15 toenail, 7 thumbnail/fingernail). (**b**) Focal areas of recurrent amplification (red) and deletion (blue) as identified by GISTIC2. Data and figure modified from Newell et al. [19]. Permission granted under a Creative Commons Attribution 4.0 International License (https://creativecommons.org/licenses/by/4.0/ accessed on 26 November 2024). Abbreviations: SMG—significantly mutated genes; SNV/Indel—single nucleotide variant/insertion–deletion; SV—structural rearrangement variants.

AM differs from other CM subtypes in terms of its relative lack of UV-induced mutations, lower tumor mutational burden (TMB), and high degree of chromosomal instability [1,2,14,15,16]. The TMB varies with the primary tumor site, with subungual tumors experiencing the greatest degree of mutational burden and some association with UV signatures, though the significance of these signatures remains cryptic [17,18,19,20]. Key implicated pathways in the pathogenesis of AM include mitogen-activated protein kinase (MAPK/ERK) and phosphatidylinositol 3-kinase (PI3K/AKT) pathways affecting cell proliferation and survival, the TERT pathway affecting telomere maintenance, the CDK4/CDKN2A pathway affecting cell cycle regulation, and the MDM2/TP53 pathway affecting apoptosis and senescence [21]. A summary of key genetic and genomic alterations in AM are shown in Figure 3.

### 3.1. Single Nucleotide Variants and Indels in the MAPK Pathway

Single nucleotide variants or insertion–deletion (indel) mutations tend to be much less common in AM compared to other CM subtypes (>18 times greater in frequency) [22]. CMs are genomically classified into four subtypes based on their mutation pattern: mutant *BRAF*, mutant *RAS*, mutant *NF1*, and triple-wild-type (TWT) [23]. Driver mutations in *BRAF, NRAS*, and *NF1* hyperactivate the MAPK/ERK pathway, leading to constitutive cell proliferation and survival. *BRAF* mutations, strongly associated with UV exposure and often a single point mutation at codon 600, cause a conformational change leading to its constitutively active state with a 480-fold increase in kinase activity [24]. Mutations in *NRAS* similarly lead to an inactivation of the intrinsic GTPase activity, leaving the RAS protein in a constitutively active, GTP-bound state and signaling for continuous cell division [25]. Finally, loss-of-function mutations in the tumor suppressor *NF1* gene impair the ability to deactivate RAS protein, promoting aberrant cell division and proliferation [26].

TWT driver mutations make up the majority (45–58%) of AM cases, with *BRAF* (10–35%), *RAS* (8–22%), and *NF1* (11–23%) driver mutations making up the rest [27]. This is in contrast with other CM subtypes, where the majority (45–50%) are *BRAF*-mutant, 30% are *RAS*-mutant, 10–15% are *NF1*-mutant, and only 5–10% tend to be TWT [27].

Among TWT mutations common to AM, activating *KIT* mutations have been commonly observed [23]. *KIT* encodes for a transmembrane receptor tyrosine kinase that binds to cytokine stem cell factor for its activation and has been shown to play critical roles in the homeostasis, survival, proliferation, and migration of melanocytes [28,29,30]. *KIT* mutations and amplifications lead to hyperactivation of its downstream MAPK/ERK, PI3K/AKT, and JAK/STAT pathways, driving cell differentiation, proliferation, and survival in the absence of ligand stimulation [30,31]. *KIT* mutations have also been linked to metastasis in various other cancers, including gastrointestinal stromal tumors (GIST), lung cancer, and acute myeloid leukemia [30,32]. *KIT* mutations are identified in approximately 3% of all melanomas, with the majority occurring in acral (36%), mucosal (39%), and chronically sun-damaged skin (CSD; 28%) melanomas [33]. Substitution mutations are most common, and the majority occur in the juxtamembrane domain of exon 11 (commonly L576P; ~70%), kinase domain I of exon 13 (~17%), and kinase domain II of exon 17 (~15%) [34,35]. Copy number amplifications of *KIT* are also frequently observed in studies as an early event in the development of AM (~27%) and mucosal melanoma (MM; ~26%) [14,33,36,37,38]. *KIT* has been associated with the migration of mutated melanocytes that results in a “field effect”, where genetically altered yet morphologically normal cells extend into a wider periphery of the invasive, in situ tumor region [39,40,41]. These field cells were reported to be associated only with lentiginous growth patterns, where *KIT* amplification may be playing a role in the broad, single-unit growth architecture of AM [40,41].

### 3.2. Structural Variants/Gene Amplification

Compared to other melanoma subtypes, AM is characterized by large structural and copy number variations rather than SNPs or indel mutations [15,17,42,43]. The rise of whole-genome (WGS) and exome sequencing studies has allowed for the identification of associated copy number gains in *KIT, CDK4, CCND1, TERT, MDM2, CLPTM1L, SKP2, NOTCH2, GAB2, YAP1, MYC, PAK1,* and *SPRED1* genes. Additionally, copy number losses have been associated with *CDKN2A, NF1, PTEN,* and *CBL,* as well as several genes involved with DNA repair (e.g., *POLE*, *PARP1*, *ATM*, *CHEK1*) and chromatin remodeling (e.g., *ARID1A*, *ARID1B*, *KMT2C*, *KMT2D*, *HDAC* alterations) [5,16,19,20,21,22,44]. *TERT* gain, *CLPTM1L* gain, and *CDKN2A* loss were statistically significant in both primary and metastatic lesions; *CDK4* and *MDM2* gains were significant only in metastatic lesions, while *PTEN* and *NF1* deletions were significant only in primary tumors [21]. In this review, we will specifically focus on copy number alterations that are most common and distinct in frequency in AM compared to other CM subtypes.

#### 3.2.1. Cell Cycle Regulation

**Figure 4:**
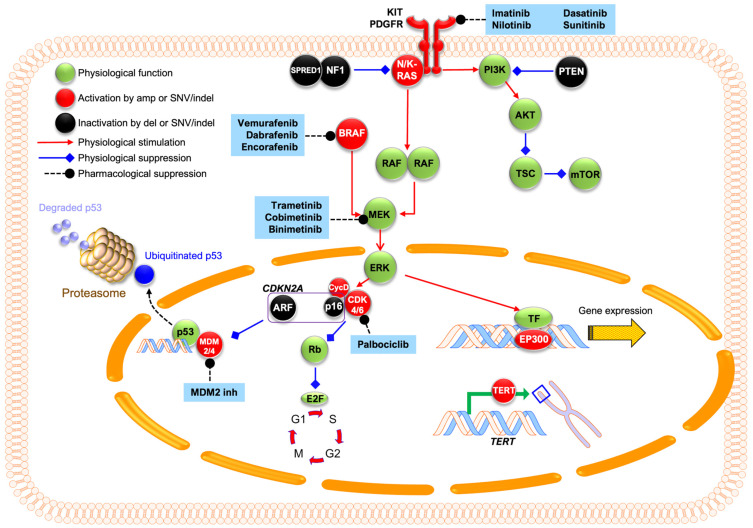
Graphic representation of common molecular pathways involved in acral melanoma and their pharmacological inhibitors. *KIT* mutations or amplifications can lead to constitutive activation of its downstream MAPK/ERK and PI3K/AKT pathways. Within the MAPK/ERK pathway, *N/K-RAS* mutations hyperactivate RAF kinases that activate downstream MEK and ERK. ERK promotes cyclin-D1 expression, which binds to CDK4/6 and phosphorylates Rb protein. This phosphorylation inactivates Rb, leading to the release of E2F transcriptional factors that drive G1–S cell cycle progression. *CDKN2A* encodes two tumor suppressors: p16^INK4A^, which inhibits CDK4/6, and p14^ARF^, which inhibits MDM2/4 and prevents p53 degradation. Loss of *CDKN2A* leads to unchecked cell cycle progression and growth. Within the PI3K/AKT pathway, *PTEN* loss leads to hyperactivation of PI3K signaling, which leads to increased mTOR signaling and drives cellular growth and protein synthesis. Dysregulation of transcriptional factor EP300 leads to altered histone acetyltransferase activity, and *TERT* promoter mutations lead to telomerase overexpression and cell immortalization. In AM, proteins with activating changes (e.g., amplification or missense mutations) are colored in red, while inactivating changes (e.g., deletions or deleterious point mutations) are colored in black. Existing inhibitors are shaded in blue.

Copy number amplifications of *CDK4* (cyclin-dependent kinase 4) and *CCND1* (cyclin-D1) are some of the most well-established aberrations in AM, the pathway of which is shown in Figure 4 [1,2,14,15]. CCND1 binds with CDK4 or CDK6 to form a complex that hyperphosphorylates the retinoblastoma protein (Rb) [45]. This leads to inactivation of Rb and release of E2F transcriptional factors, which drive the cell cycle transition from the G1 to S phase. Although *CCND1* amplification is most common, amplification of other D-type cyclins *CCND2* and *CCND3* has also been occasionally observed [20].

*CDKN2A* is a tumor suppressor gene that encodes protein products p14^ARF^ and p16^INK4A^ and is observed to be lost in AM. p14^ARF^ is an inhibitor of MDM2 ubiquitin ligase (E3) that degrades tumor suppressor protein p53, thereby stabilizing p53, while p16^INK4A^ inhibits CDK4/6 and prevents the phosphorylation of Rb protein, thereby arresting the cell cycle at G1 [46,47]. Loss of *CDKN2A* disrupts these protein functions, leading to sustained cell cycle progression and uninhibited cell growth. WGS studies have suggested that TWT melanomas, commonly of the AM subtype, are more likely to have focal amplifications of *CCND1, MDM2*, *KIT,* and *KRAS* [22], as well as *CDK4* and *CDK6* [19], than non-TWT melanomas.

Various studies have demonstrated the promise of CDK4/6 pathways to be a target for treatment. In a study by Yeh et al., activating mutations involving the CDK4/6 pathway were identified in 62.3% of 122 AM cases [48]. Co-amplification of *MDM2* was common in *CDK4*-amplified cases [48]. Another WGS study by Newell et al. demonstrated *CDK4*, *CDK6,* and *CCND1* amplifications in 53% of AM tumors [19]. In a retrospective study by Kong et al., 82.7% of 514 AM samples had aberrations of *CDK4* gain, *CCND1* gain, or p16^INK4a^ loss [49]. The same study demonstrated anti-tumor effects of CDK4/6 inhibitors in *CDK4*-gain AM cell lines and patient-derived xenograft mouse models. These results paved the way for clinical trials of CDK4 inhibitors for AM patients, which are discussed in a later section.

#### 3.2.2. TERT: Copy Number Increases and Promoter Mutations

*TERT* (telomerase reverse transcriptase) aberrations have been well-documented to play a role in AM, though their reported frequency and significance have varied. Activating *TERT* mutations and copy number gain lead to increased telomerase expression, allowing cells to continue proliferating and acquire immortalization [50]. *TERT* promoter mutations are often UV-associated and commonly occur as an early event in CM to drive overexpression [22,51,52,53]. In AM, several studies have identified a lower frequency of *TERT* promoter mutations (9.3%) [54] and amplifications (10.7%) [48], whereas a study by Liang et al. identified *TERT* translocations, amplifications, and mutations in up to 41% of cases [21]. Recently, Wang et al. sequenced AM samples at different stages of progression and identified clusters of copy number transitions (termed hailstorms) predominantly involving chromosomes 5p, 11q, 12q, and 22q even in the earliest stages of melanoma progression [55]. Among these, *TERT* alterations were the most common, present in 70.3% of samples most frequently as high-level amplifications and occurring at the earliest progression stages. This early hyperactivation of telomerase maintenance suggests underlying mechanisms of telomere crisis and/or erosion arising early in melanoma development, possibly via chromatids with short telomeres fusing together, inducing breakage-fusion-bridge cycles and chromothripsis [55]. Another proposed mechanism involves physical force-induced DNA breaks leading to early telomerase activation for chromosomal healing. Acral sites tend to be pressure-bearing anatomical regions, and physical force has been implicated in breakage-fusion-bridge cycles triggering chromothripsis [55]. Chromosomal healing in this case would entail capping double-stranded DNA breaks with new telomeres, which aligns with findings by Newell et al. that showed longer telomeres in AM than other CMs [17].

#### 3.2.3. MDM2 Amplification and p53 Alterations

AM is less driven by alterations in the TP53 pathway compared to other cancers [22,48], but loss-of-function mutations and structural variants of *TP53* have been reported as late events in AM [56]. The *TP53* gene encodes the tumor suppressor protein p53, which prevents the proliferation of genetically compromised cells by regulating cell cycle arrest and apoptosis [46]. P53 is negatively regulated by MDM2, a ubiquitin ligase that, along with its homolog MDM4, targets p53 for degradation and suppresses its transcriptional activity [46]. *MDM2* is commonly reported to be amplified in AM, which may result in excessive suppression of p53 and contribute to unchecked cell proliferation [19,44,55]. *MDM2/4* and *EGFR* amplifications have been associated with hyper-progression of tumor growth following ICIs in certain cancers [57]. ICI treatment outcomes of metastatic AM and MM with *MDM2/4* or *EGFR* amplifications have been studied; the association between hyper-progression and *MDM2/4* or *EGFR* amplification in these AM/MMs was not statistically significant [58].

### 3.3. Other Recent Genetic Findings

Recent sequencing studies have identified novel driver genes that were not previously described in AM. Genomic analysis of AM and MM proposed *PTPRJ* (tumor suppressor gene mutations and homozygous deletion) as well as *FER* and *SKP2* (oncogene amplification) as potential novel driver genes [44]. Genes of the SWI/SNF complex (a subfamily of ATP-dependent chromatin remodeling complexes), including *SMARCA2*, *SMARCA4*, *SMARCD1*, *ARID1A*, *ARID1B*, *ARID2*, and *PBRM1*, were altered in 13.8% of samples [44]. The study additionally identified infrequent alterations of *CIC* and *LZTR1* as well as pathogenic germline mutations in *MITF, PTEN, ATM,* and *PRKN* [44].

### 3.4. Markers for Metastasis and Prognosis

Recent work has underscored genetic markers that may drive metastasis and induce worse prognosis in AM. Farshidfar et al. reported amplifications of 22q11.21 that were enriched in metastatic AM tumors, wherein *LZTR1* is a key gene and often co-amplified with *CRKL* [16]. Silencing of *LZTR1* arrested cell proliferation and induced apoptosis in melanoma cell lines (both AM and CM) irrespective of RAS or MAPK activity. On the other hand, overexpression of *LZTR1* in normal melanocytes induced processes of malignant transformation and metastasis, such as spheroid formation and anchorage-independent growth [16].

*MITF* is a melanocytic lineage-specific transcription factor that has been heavily implicated in malignant melanoma and associated with poor prognosis and metastasis [59,60]. Pathogenic germline mutations and amplifications of *MITF* have been associated with AM in several studies [14,44,48,55]. Recently, a study by Wei et al. proposed a *MITF*-mediated shift towards fatty oxidation (FAO) in *MYC+* cells as an underlying mechanism for AM lymph node (LN) metastasis [61]. FAO genes (*MYC, MITF*, and *CTNNB1*) were enriched in the *MYC*+ cell cluster, and overexpression of *MITF* was associated with greater LN metastasis in mouse footpad melanoma. The application of etomoxir, an FAO inhibitor, demonstrated the ability to limit mouse footpad LN metastasis but did not change the primary tumor size, suggesting its potential to target *MITF*-mediated metastasis [61].

In Taiwanese patients, cell cycle aberrations (*CDK4/6, CCND1/2*, and *CDKN2A*) and gains in anti-apoptosis genes (*BIRC2, BIRC3*, and *BIRC5)* have been associated with inferior MSS compared to those without such aberrations (4.2 vs. 15.1 years; 4.0 vs. 12.6 years) [62]. Multivariate analysis pointed to cell cycle aberrations and LN metastasis as independent prognostic markers of MSS [62]. Additionally, *AURKA* copy number gain has been linked to poorer prognosis of AM [14], with both overall survival (OS) and progression-free survival (PFS) being significantly shorter compared to those with normal copy numbers (OS: 48.5 vs. 59.8 months; PFS: 13.3 vs. 30.0 months) [63]. Relapse-free survival (RFS) following treatment with high-dose interferon (HD-IFN) therapy was also found to be shorter in those with *AURKA* copy gain, suggesting that *AURKA* gain could be a poor prognostic factor in treatment [63]. Other proposed prognostic markers have included *NF1* mutations, *TERT* amplifications, and certain mutational signatures [14]. *TERT* copy gain in particular has been found to be a significant predictor of RFS in AM patients receiving HD-IFN, although it was not a significant prognostic marker of OS [64].

### 3.5. Anatomical Specificity

It has been hypothesized that even amongst cases of AMs, the genetics of tumorigenesis may vary based on the anatomical location. For instance, subungual/interdigital AM has been associated with greater *CDK4* and *CCND1* gains, fewer *BRAF* mutations, and less likelihood of being a superficial spreading histologic subtype compared to volar AM [5]. This suggests a genetic driving mechanism that may differ based on the site of AM tumorigenesis.

Recent studies have begun to identify several genes that may be implicated in the unique anatomical sites of AM. Weiss et al. identified *CRKL* and *GAB2* amplifications to be highly enriched and often co-occurring with *TERT* amplifications and *NF1* losses [65]. Overexpression of *CRKL* in zebrafish models drove melanocyte tumorigenesis and enrichment to fin compartments, analogous to hand and feet sites in humans, thereby implicating *CRKL* amplification as a critical contributor to acral specificity. The elimination of *CRKL* removed this anatomic specificity. *GAB2*, *TERT*, and *NF1* aberrations alone were not sufficient to drive acral tumorigenesis [65].

AM has long been associated with development in plantar regions that are UV-protected but experience high mechanical stress, frequently occurring on the heel [66,67]. In a similar vein, much of the mechanical stress while standing or walking has been found to concentrate on the heel and forefoot [68,69]. Although AM and MM are thought to be less likely associated with TP53, PTEN, or RB1 pathway lesions [22], *PTEN* alterations especially related to plantar surfaces are of note. *PTEN* is a tumor-suppressor gene that plays a role in cell differentiation via the PI3K-AKT pathway. *PTEN* mutations associated with AM were more commonly frameshift indel mutations and tended to be absent in subungual melanomas [19]. In a recent retrospective Korean study, *PTEN* promoter hypermethylation was found to be present in 25% of AM patients and was associated with greater Breslow thickness and ulceration rates, specifically in the heel, forefoot, and hallux [70]. The authors suggested an epigenetic involvement in tumorigenesis that may be linked to long-term mechanical stress. Moreover, another study of Korean melanoma samples, 50% of which were acral, revealed hypermethylation of the *PTEN* promoter to be a significant adverse prognostic marker in melanoma [71]. Such work prompts investigation into the mechanisms underlying these unique alterations that become enriched in weight-bearing regions.

### 3.6. Mechanical Stress and Chromothripsis

**Figure 5:**
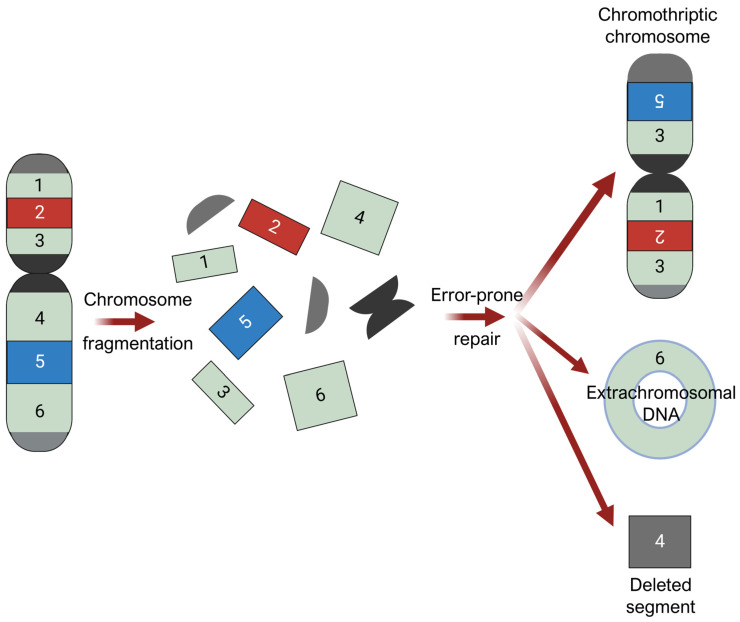
Chromothripsis. Chromosomal fragmentation generates multiple double-stranded DNA breaks, followed by error-prone repair processes that reassemble the resulting DNA fragments in a random order and orientation. Chromosomal segments that fail to reintegrate into chromosomes may self-ligate, forming extrachromosomal DNA (ecDNA) structures, or may be eliminated, resulting in deletions. Figure was prepared using BioRender.

There is emerging speculation that chromothripsis contributes to AM development in weight-bearing areas under high mechanical stress [72]. Chromothripsis is a massive chromosomal rearrangement that arises from the shattering and disorganized reassembly of large chromosomal regions in one catastrophic event, as demonstrated in Figure 5 [19,22,72,73,74,75]. It is thought to arise from breakage-fusion-bridge cycles, where chromosomal breakage and/or telomere crisis leads to telomeres fusing together to form dicentric chromosomes [76,77], and from mitotic defects, where mis-segregated chromosomes during mitotic exit are sequestered into micronuclei formation [73]. Micronuclei are prone to structural instability, such as defective assembly and rupturing of their nuclear envelopes [78,79,80]. When the nuclear envelope is disrupted, DNA within micronuclei is exposed to cytosolic nucleases, invasion of endoplasmic reticulum, and other DNA stressors, triggering massive damage and resulting in chromosome shattering [81]. In subsequent cell division cycles, these shattered chromosomal fragments can undergo chaotic, error-prone reassembly, resulting in genomic rearrangements of random order and orientation. Chromosomal segments that fail to reintegrate into chromosomes may self-ligate, forming extrachromosomal DNA (ecDNA) structures, or may be eliminated, resulting in deletions.

Chromothripsis is widely considered an early event in cancer evolution [74,82]. In AM, regions of *CCND1* amplification contained almost no mutations that seemed to predate the amplification event, whereas hundreds to thousands of mutations were amplified in CM [74]. This alludes to the particularly early nature of chromothripsis in AM, preceding most somatic point mutations.

Macroscopic mechanical stress is hypothesized to disrupt nuclear envelope integrity and induce micronuclei membrane rupture implicated in chromothripsis, activating *YAP* (Yes-Associated Protein). *YAP* is a mechanosensitive transcriptional cofactor that senses and mediates mechanical cues, including matrix stiffness as well as cell geometry, adhesion, and stretching [72,83]. Matrix stiffening activates *YAP*, which also regulates the expression of cytoskeletal regulators and can promote greater matrix stiffening in a feed-forward manner [84]. *YAP* has been strongly implicated in cancer initiation and growth in a variety of malignancies [85] and has also been associated with melanoma metastases [86,87].

In a recent study by Seo et al., mouse footpads were implanted with melanoma cells and assessed for nuclear membrane instability [72]. An increase in micronuclei and nuclear membrane rupture was observed in the marginal, weight-bearing regions of the plantar melanoma compared to its center. Additionally, proinflammatory genes of the cGAS-STING pathway, known to be implicated in tumor progression, were elevated in footpad tumors compared to non-weight-bearing trunk tumors. *YAP*-activated sites correlated with greater degrees of DNA damage and nuclear membrane rupture in both human plantar melanoma and mouse models, while pharmacological inhibition of *YAP* in mouse footpads led to a reduction in melanoma proliferation. Accordingly, the authors proposed that macroscopic mechanical stress might increase *YAP* activation via nuclear translocation, potentially playing a critical role in the frequent tumorigenesis of AM in weight-bearing regions [72]. Furthermore, *YAP* has been suggested to play a role in disseminating melanoma cells via negative durotaxis (directed cell migration towards softer environments) [88].

The final complex chromosomal rearrangement discussed in this review involves “tyfonas,” which are regions of high-density copy number junctions and fold-back inversions enriched with protein-coding fusions and breakend hypermutations [89]. Tyfonas are thought to arise during the transient ecDNA phase, where progressive self-assembly and amplification of ecDNA fragments result in high-junction copy number structures characterized by the aforementioned alterations. Hadi et al. found that tyfonas were associated with *MDM2* and *CDK4* amplifications and commonly associated with AM (40%) but rarely with CM (<2%) [89]. The specificity of tyfonas for the AM tumor type was even greater than that of amplified chromothripsis, suggesting tyfonas to be a defining mechanism for genomic instability in AM. Enrichment of fusion transcripts in tyfonas was suggested as an alternative source of neoantigens, potentially contributing to the mechanism of immunotherapy for AM [89].

## 4. Current Therapies and Clinical Implications

Despite advances in immunotherapy/ICI and targeted therapies for malignant melanoma, AM has demonstrated lower efficacy from such therapies, likely due to a lower mutational burden compared to other CM subtypes [90]. Current guidelines are as follows. For localized, resectable disease, surgical resection is the standard of care [10]. Subungual AM often involves amputation; however, recent studies have suggested less invasive techniques such as wide local excisions and Mohs microscopic surgery to provide statistically similar local control and recurrence rates as amputations [10,91,92]. For unresectable and advanced disease, various systemic therapy options are described below.

### 4.1. Immune Checkpoint Blockade

Combination immunotherapy of anti-PD-1 therapy (e.g., nivolumab, pembrolizumab) with anti-CTLA-4 therapy (e.g., ipilimumab) is the recommended treatment to date. Retrospective analysis of PD-L1 expression in Japanese melanoma patients demonstrated lower expression levels in AM than CM (13.6% vs. 44.7%; *p* = 0.014) [93]. Similarly, a retrospective study of nivolumab in a Japanese cohort demonstrated worse average objective response rates (ORRs) for AM and MM in comparison to CM (19% and 21% vs. 43%) [94]. This trend persisted in AM cases with visceral metastasis (ORR: AM/MM 13% vs. CM 42%; *p* = 0.028) [94]. One possible explanation is that AM is associated with a lower and more heterogenous immune infiltrate than CM, which may contribute to AM’s relatively poor response to immunotherapy [95,96,97]. Single-cell RNA sequencing (scRNA-seq) of AM has revealed lower levels of natural killer cells, effector CD8+ T cells, and gamma delta T cells compared to non-acral CM, with AM samples demonstrating high levels of interpateint heterogeneity [96]. Moreover, another study found greater levels of regulatory T cells (Tregs) in AM compared to CM, which may contribute to ICI resistance due to Tregs inhibiting immune cell-mediated tumor cell death [97].

Despite this, recent ICI studies of advanced AM/MM patients have demonstrated some response, particularly to anti-PD-1 therapy [10,94,98,99,100,101,102,103]. One multicenter study of AM/MM patients treated with nivolumab or pembrolizumab reported an ORR of 32% (95% CI: 15–54%), median PFS of 4.1, and median OS of 31.7 months in AM [90]. However, studies from countries such as China and Japan, where AM makes up a greater proportion of melanoma patients, have reported overall lower rates of anti-PD-1 efficacy, with ORRs between 14 and 26.7%, median PFS between 2.8 and 6.6 months, and median OS between 14 and 18.1 months [10,94,98,99,100,101,102]. Combination therapy of anti-PD-1 and anti-CTLA-4 has been found to be more effective than monotherapies (ORRs ranging 40–43% vs. 15–26%) [104,105,106]. The CheckMate 172 phase II study of nivolumab given to patients who progressed during or after ipilimumab treatment demonstrated a median OS of 25.8 months (95% CI: 15.1–30.6 months) for AM, with an 18-month OS rate of 59.0% (95% CI: 44.2–71.1%) [103]. Combination therapy was especially more effective than monotherapy in nail apparatus melanomas (ORR: 61% vs. 10%, *p* < 0.001; PFS: 6.4 vs. 3.8 months, *p* = 0.1) [104]. Of note, a recent study comparing combination regimens of nivolumab plus ipilimumab (data from phase III CheckMate 067) versus nivolumab plus relatlimab (data from phase II/III RELATIVITY-047) reported a preference for the nivolumab plus ipilimumab regimen for AM tumors (HR 1.42; 95% CI: 0.69–2.93) [107].

### 4.2. Adjuvant Therapies

In addition to more traditional HD-IFN therapy, ICIs are increasingly being considered for adjuvant therapy following surgical resection of advanced melanoma. However, the benefit of ICIs versus HD-IFN in AM remains unclear. A retrospective study in Chinese patients found no significant benefit in RFS or distant metastasis-free survival (DMFS) with adjuvant anti-PD-1 treatment compared to adjuvant HD-IFN in AM patients, although such benefits were observed in CM patients [108].

Recent findings point to a lower efficacy of adjuvant therapy in AM compared to other CM subtypes. When the efficacy of adjuvant therapy (including anti-PD-1 agents and BRAF/MEK inhibitors) was analyzed in a Japanese study, the 3-year time-to-relapse (TTR) was significantly worse in AM patients compared to non-acral CM patients following adjuvant therapy (HR 0.56; 95% CI: 0.34–0.92; *p* = 0.021) [109]. Furthermore, a nationwide Dutch study of melanoma patients who received anti-PD-1 adjuvant therapy after complete resection found that AM patients had a significantly lower median RFS than CM patients (14.8 vs. 37.4 months; *p* = 0.002) as well as a higher risk of recurrence (HR 1.53; *p* = 0.019) [110]. AM patients also demonstrated a worse two-year DMFS (64.5% vs. 79.7%; *p* = 0.050) and two-year OS (71.5% vs. 84.3%; *p* = 0.027) than CM patients [110].

Despite such limitations, some evidence supports the potential role of anti-PD-1 adjuvant therapy in AM. A Chinese study of 174 stage III melanoma patients, 67.7% (n = 118) of whom were of the AM subtype, reported that anti-PD-1 adjuvant therapy improved disease-free survival (DFS) compared to HD-IFN or observation-only treatment in the overall cohort (*p* = 0.039) [111]. Interestingly, no survival benefits were observed in patients harboring *BRAF*, *NRAS*, or *KIT* mutations. In contrast, wild-type patients demonstrated better DFS when treated with anti-PD-1 adjuvant therapy compared to HD-IFN or observation-only treatment (*p* = 0.003) [111]. Further work needs to be conducted to accurately define the role of immunotherapy as an adjuvant therapy for postoperative AM.

A relatively recent advancement involves oncolytic therapies such as T-VEC, a modified herpes simplex virus type 1 therapy that was FDA approved for unresectable melanoma in 2015 [112]. T-VEC induces tumor-specific T-cell responses while expressing human granulocyte-macrophage colony-stimulating factor (GM-CSF) that aids in the recruitment and priming of immune cells for enhanced anti-tumor activity [112]. Since then, neoadjuvant oncolytic virus orienX010 (ori) and neoadjuvant PD-1 inhibitor toripalimab (tori) have entered phase Ib clinical trials for stage III/IV AM patients [113]. So far, the neoadjuvant ori and tori regimen (paired with surgery and adjuvant tori) is showing initial promise for AM, with 77.8% pathological and 36.7% radiographic response, 1- and 2-year RFS rates of 85.2% and 81.5%, and predominantly grade 1–2 adverse events (AEs).

### 4.3. Molecular Therapies

#### 4.3.1. c-Kit Inhibitors

A number of KIT inhibitors have been developed and are in clinical trials to target unresectable melanomas, including imatinib, nilotinib, dasatinib, and sunitinib [30,31]. The pooled ORR for KIT inhibitors in AM patients across 19 single-arm studies was reported to be 22% (95% CI: 14–30%) [31]. The following clinical trials were selected based on the most recent and advanced stage of trial for each drug, with imatinib and nilotinib showing the most promise.

Imatinib was the first tyrosine kinase inhibitor (TKI) studied that was initially developed to inhibit BCR-ABL and PDGFR. It is currently used as a first-line treatment for conditions like chronic myeloid leukemia and advanced GIST [30,114]. Most recently in 2022, a pooled analysis of 130 *KIT*-altered melanoma patients (AM n = 6) demonstrated an ORR of 25%, PFS of 2.7 months, and OS of 21.8 months in AM patients [115]. A *KIT* mutation in exon 11 or 13 was associated with a longer median PFS than was a mutation in exon 17, though the difference was not statistically significant (4.3–4.5 vs. 1.1 months) [115]. In 2019, a retrospective study of 78 patients (AM n = 33) reported an overall PFS of 4.2 months (95% CI: 1.9–6.4 months) and OS of 13.1 months (95% CI: 9.6–16.7 months) [116]. Similarly, imatinib’s performance varied based on the *KIT* mutation being in exons 11 or 13 (60.2%) or exons 9, 17, or 18 (29.5%), though the results were not statistically significant (ORR: 24.4% vs. 19.4%; disease control rate: 66.7% vs. 54.8%) [116]. The most common AEs were nausea, fatigue, hyperglycemia, and vomiting [117].

Nilotinib is a more potent second-generation TKI demonstrating potential promise for *KIT*-mutated melanoma [114,118]. Most recently in 2024, the multicenter, single-arm NICAM phase II trial evaluated nilotinib in 26 *KIT*-mutated melanoma patients (AM n = 6) [119]. The ORR was 19% (95% CI: 7–39%) at 12 weeks, and 25% of patients were alive and progression-free at 6 months. In acral tumors specifically, the PFS was 2.3 months and the OS was 5.1 months. A total of 64% of the overall patients reported AEs of grade 3 or higher, with the most common overall AEs being fatigue, nausea, and constipation. In a pooled study, imatinib was associated with a slightly higher ORR (27%; 95% CI: 14–42%) than nilotinib (22%; 95% CI: 11–34%) [31].

Dasatinib is also a second-generation TKI that targets KIT, Src family kinases, PDGFR, and BCR-ABL [120]. The phase II ECOG-ACRIN E2607 trial assessed dasatinib in 73 patients with acral, mucosal, and CSD subtypes across stage I (*KIT*+ and wild-type; n = 51) and stage II (*KIT*+ only; n = 22) [120]. There was a partial response (PR) of 3/51 (5.9%; 90% CI: 1.6–14.5%) in stage I and of 4/22 (18.2%; 90% CI: 10.4–46.6%) in stage II. Three out of four patients who achieved PR in stage II had melanomas of the *KIT*+ acral subtype. Stage II ended early due to slow accrual. Toxicity was relatively high, with grade III AEs occurring in 44% of patients and dasatinib being discontinued in 12% of patients [120]. Another phase II trial of dasatinib similarly demonstrated low response rates of only 5% (95% CI: 1.5–18.1%), concluding that dasatinib has minimal efficacy and is poorly tolerated in advanced melanoma patients [121].

Sunitinib, which inhibits KIT and vascular endothelial growth factor receptors, also demonstrated a limited response in advanced melanoma patients [30]. A preliminary 2012 study evaluated sunitinib in twelve patients, two of whom had melanomas of the AM subtype and were evaluable for response (one with stable disease and one with progressive disease following sunitinib) [122]. In a 2015 phase II study of sunitinib in 52 patients with metastatic AM or MM, 4 patients had confirmed PR (8%; 95% CI: 2–19%) and 23 patients experienced disease control (44%; 95% CI: 30–59%) [123]. There was no significant difference in response between patients with and without *KIT* mutations (ORR 7.7% vs. 9.7%; OS 6.4 vs. 8.6 months). Overall, there were no prolonged responses, and the toxicity was deemed high.

#### 4.3.2. BRAF/MEK Inhibitors

Studies regarding the efficacy of BRAF/MEK inhibitors specific to AM are sparse; targeted therapies such as BRAF/MEK inhibitors are often not elected in AM treatment unless the AM is *BRAF V600E/K* mutated [10]. As mentioned previously, AM generally demonstrates a lower mutational burden and carries greater heterogeneity in its *BRAF* mutations [124]. For *BRAF*-mutant AM cases, BRAF/MEK inhibitors show promise as a treatment. East Asian countries have reported ORRs of 38.1% [125], 64.3% [126], 78.9% [127], and 83.3% [128] for BRAF/MEK inhibitors, although each study only had 10–30 AM patients and thus cannot be used to conclude broader efficacy [10,129].

#### 4.3.3. CDK4/6 Inhibitors

To date, palbociclib is the only CDK4/6 inhibitor to have published clinical trial results for advanced AM [130]. CDK4/6 inhibitors (e.g., palbociclib, ribociclib, abemaciclib) recently emerged as promising therapeutics for hormone receptor-positive, *HER2*-negative breast cancer that harbors similar CDK4/6 pathway dysregulation as AM [131]. When tested in AM cell lines and xenograft models, CDK4/6 inhibitors demonstrated preclinical anti-tumor effects, targeting aberrant cell proliferation [49]. Preclinical studies have also suggested that CDK4/6 inhibitors may increase tumor cell immunogenicity and are being explored as candidates for combination therapy with immunotherapies [132]. The phase II clinical trial (NCT03454919) of palbociclib demonstrated preliminary efficacy and acceptable safety in advanced AM patients with *CDK4* gain, *CCND1* gain, and/or *CDKN2A* loss [133]. A total of 20% (3/15) of AM patients experienced tumor shrinkage at 8 weeks, with a median PFS of 2.2 months (95% CI: 1.9–2.5 months) and median OS of 9.5 months (95% CI: 5.7–13.4 months). Safety was considered acceptable, with the majority of AEs being grade I–II. Interestingly, those who did not respond to palbociclib often harbored significant *JAK2* deletions and *SH2B3* amplifications. Another CDK inhibitor, dinaciclib, is currently in clinical trials for stage IV melanoma patients, including those with AM, but the results have yet to be published (NCT00937937) [129].

A recent study by Jagirdar et al. has attributed resistance to CDK4/6 inhibitors in AM to hyperactivation of the MAPK pathway and elevated *CCND1* expression [134]. The study reported enhanced preclinical efficacy of CDK4/6 inhibitors when combined with MEK/ERK inhibition in both treatment-naive and resistance-acquired xenograft models, supporting the investigation of a CDK4/6 inhibitor combined with a MEK inhibitor as a potential therapy option. Future clinical trials are necessary to determine the efficacy of CDK4/6 inhibitors for treatment of advanced AM.

### 4.4. Clinical Implications

As noted previously, AM is associated with overall worse prognoses and greater rates of acquired resistance compared to other CM subtypes. AM tumors are also often *BRAF* wild-type and have low tumor infiltration, making treatment with existing BRAF/MEK inhibitors and immunotherapies particularly challenging. Combination therapy is a promising approach to address resistance and low response rates by targeting multiple complementary oncogenic pathways without a dependence on one. This highlights the need for novel therapeutics that exploit distinct oncogenetic pathways to broaden treatment options. In addition to BRAF/MEK inhibitors and ICIs, emerging candidates such as CDK4/6 inhibitors in clinical trials allow targeting of distinct pathways to minimize the risk of resistance [129,130]. Novel targeted therapeutics in combination with ICIs may further sensitize the tumor to therapy, possibly enhancing immunogenic recognition and infiltration especially critical in low-TMB tumors such as AM. This precision medicine-driven approach helps ensure that targeted therapies are properly matched to the molecular drivers of disease, helping maximize treatment efficacy while minimizing resistance in AM patients.

Finally, a deeper genetic understanding of AM can improve risk stratification and prognostication in clinical practice. Tumor sequencing enables the identification of patient-specific mutations, facilitating the use of therapeutics that attack vulnerabilities unique to individual AM tumors. Moreover, emerging prognostic and metastatic markers, such as *LZTR1, MITF, AURKA*, *TERT*, and cell cycle aberrations, warrant validation to support early prognostication and enhance personalized treatment regimens. A more refined genetic understanding also enables early screening and preemptive measures to reduce risk. For example, insights into chromothripsis can help guide lifestyle modifications, such as recommending padded footwear and avoiding repeated trauma associated with DNA damage in at-risk populations. Preventative and prognostic measures are especially important for darker-skinned individuals, who disproportionately face higher prevalence rates and delayed diagnoses. Novel genetic and molecular insights hold the power to refine prognostication and enhance treatment of AM at unprecedented levels of personalization.

## 5. Conclusions and Future Directions

AM is distinct from other CM subtypes both in its tumorigenesis and clinical implications. Its low mutational burden, limited immune filtration, and marked chromosomal instability all contribute to its resistance to current therapies. Recent sequencing studies have elucidated key pathways involved in tumorigenesis, metastasis, telomere/chromosomal fusion, and response to mechanical stress that can shape the future of AM management. These findings highlight a need to deepen our genetic and molecular understanding to better target this challenging subtype of melanoma.

Some areas that remain unanswered include the role of chromothripsis in the pathogenesis of AM. While chromothripsis has been linked to mitotic errors, telomere crisis, and DNA damage, the exact cellular and molecular triggers for its formation in AM are unclear. What makes acral melanocytes or certain chromosomal regions more susceptible is not well understood. The specific DNA repair mechanisms (e.g., non-homologous end joining, microhomology-mediated end joining) involved in the reassembly process need further elucidation. The factors influencing the choice of repair pathway are also unknown. While chromothripsis is primarily studied in cancer and developmental disorders, its potential occurrence and role in normal pigment cells or aging processes are largely unexplored. Lastly, the impact of chromothripsis on chromatin structure, gene regulation, and epigenetic modifications is poorly understood, as is the role of chromothripsis in immune evasion or immune system recognition in cancer.

Despite the variety of studies delineating changes across AM genomes, there is still a critical need for better therapies. A deeper genetic and molecular understanding allows us to refine patient prognostication and deploy therapies tailored to an individual’s tumor makeup. Metastatic AM represents a dwindling subgroup of treatment-resistant melanomas that need a viable therapeutic approach. With a more detailed understanding of AM etiology, more personalized and effective strategies might be possible in due time.

## Data Availability

Not applicable.
